# Radiomics-based Prognosis Analysis for Non-Small Cell Lung Cancer

**DOI:** 10.1038/srep46349

**Published:** 2017-04-18

**Authors:** Yucheng Zhang, Anastasia Oikonomou, Alexander Wong, Masoom A. Haider, Farzad Khalvati

**Affiliations:** 1 Dept. of Medical Imaging, Sunnybrook Research Institute, University of Toronto, Toronto, ON, Canada; 2 Dept. of Systems Design Engineering, University of Waterloo, Waterloo, ON, Canada

## Abstract

Radiomics characterizes tumor phenotypes by extracting large numbers of quantitative features from radiological images. Radiomic features have been shown to provide prognostic value in predicting clinical outcomes in several studies. However, several challenges including feature redundancy, unbalanced data, and small sample sizes have led to relatively low predictive accuracy. In this study, we explore different strategies for overcoming these challenges and improving predictive performance of radiomics-based prognosis for non-small cell lung cancer (NSCLC). CT images of 112 patients (mean age 75 years) with NSCLC who underwent stereotactic body radiotherapy were used to predict recurrence, death, and recurrence-free survival using a comprehensive radiomics analysis. Different feature selection and predictive modeling techniques were used to determine the optimal configuration of prognosis analysis. To address feature redundancy, comprehensive analysis indicated that Random Forest models and Principal Component Analysis were optimum predictive modeling and feature selection methods, respectively, for achieving high prognosis performance. To address unbalanced data, Synthetic Minority Over-sampling technique was found to significantly increase predictive accuracy. A full analysis of variance showed that data endpoints, feature selection techniques, and classifiers were significant factors in affecting predictive accuracy, suggesting that these factors must be investigated when building radiomics-based predictive models for cancer prognosis.

The current clinical workflows generate thousands of images per patient making it impractical for clinicians to study all the images. Moreover, human interpretation of medical images is inherently biased and prone to fail in discovering the entirety of potentially informative imaging data. As a new field of study, radiomics aims to discover and translate this un-decoded information in medical images^1^. Radiomics is defined as the extraction of a large amount of quantitative features from medical images^2^. By capturing the entirety of tumor site and the ability of extracting information from 3D images, radiomics has the distinct advantage of assessing tissue heterogeneity, a well described phenomenon in cancer analysis with varying cell phenotypes. This is in contrast to other clinical procedures such as biopsy where only a small fraction of tumor is sampled with the significant chance that the index tumor is entirely missed^3^ leading to misinterpretations and non-optimal clinical decisions.

Radiomic features offer comprehensive and quantitative measurements of tumors through 3D images including texture, intensity, heterogeneity, and morphology information allowing a comprehensive analysis of tumor phenotype^1^. Recent studies have found that radiomic features may have significant associations with clinical outcomes and gene-expression levels^4^,^5^,^6^,^7^,^8^,^9^. These features can also be used to develop diagnosis or prognosis models that may serve as a tool for personalized diagnosis and clinical decision support systems.

Radiomics-based prognosis analysis pipeline constitutes several stages. Figure 1 shows a typical pipeline for radiomics analytics. First, raw images are pre-processed to annotate regions of interest (ROIs) such as cancerous regions (tumors). This is usually done by contouring the ROIs manually by clinicians or automatically via segmentation algorithms^3^. Next, a large number of quantitative imaging features is extracted from these ROIs. In the next step, endpoint data (i.e., clinical outcome such as disease recurrence) is analyzed to guide the construction of predictive models which involves feature reduction and outcome classification.

Although previous studies have found several radiomic features having significant association with clinical outcomes including survival or recurrence for different cancer types, each individual feature could only explain a small amount of variation in the outcomes^7^. In addition, the traditional testing methods are prone to lead to the multiple testing problem, increasing the false positive rate (type one error) if not corrected, or increasing the false negative rate (type two error)^10^,^11^. Setting critical P value as 0.05, we expect to see 5 significant results from 100 testing using random data. Hence, in radiomics analytics, since the number of features is usually large (e.g., 100), the impact of multiple testing problems is significant and perhaps unavoidable. Furthermore, with a large number of features, radiomics studies generally have small sample sizes, leading to the “Large P, small N” problem, where the number of features is much larger compared to the sample sizes^11^. Moreover, the clinical outcomes are usually unbalanced, which is not optimal for model training. These negatively affect the prediction accuracy of prognosis models, which need to be addressed when building an efficient radiomics-based prognosis model.

In this study, we investigate the predictive performance of the combinations of 5 unfiltered feature reduction techniques and 8 different classifiers applied to quantitative CT feature of a dataset of Non-small Cell Lung Cancer (NSCLC) patients with 3 clinical outcome namely recurrence, death, and recurrence-free survival. To address the unbalance dataset problem, 4 subsampling methods were also investigated. We aim to determine an optimal configuration of radiomics-based prognosis analytics in terms of feature selection, subsampling, and predictive models that yields the best prognosis accuracy for the disease and thus, potentially enhancing the applicability of radiomics analytics in clinical practice.

## Methods

### Dataset

This retrospective study was approved by the Research Ethics Board of Sunnybrook Health Sciences Centre and all methods were performed in accordance with the guidelines and regulations of this ethics board. 112 NSCLC patients with mean age 75 years who underwent stereotactic body radiotherapy (SBRT) were included in this study. CT scans, manual contours, and clinical outcomes were available for all included patients. Patients were followed-up with CT of the chest and abdomen every 4 months for the first 3 years after SBRT and every 6 months thereafter. Local and lobar recurrences were assessed for each pulmonary lesion treated. Regional, distant failure and overall survival were calculated based on each patient treated. Recurrence, death, and recurrence-free survival were calculated based on the status of patients at the end of the 3-year follow-up.

The delineation of the tumors was performed manually consistently by a thoracic radiologist with 14 years of experience in thoracic imaging using ProCanVAS, an in-house developed computer-aided diagnosis tool for cancer analysis^12^. Each lesion was contoured on every sequential slice that was visible and the largest ROI for each patient was selected as the representative cross section of the lesion. In total, 2159 ROIs were assessed and the lesions had a cross section mean size of 242 mm^2^. Thirty radiomic features (11 first order features, and 19 second order features) were extracted from the ROIs with maximum size for each patient. Table 1 lists details of the radiomics features^13^,^14^.

### Feature reduction and predictive models

A large number of quantitative features can be extracted from medical images. However, many of these feature may be simply noise, or highly correlated with each other. Feature reduction is necessary to select a subset of useful and unique features, increasing the prediction accuracy and minimizing the computational cost. The feature reduction procedure can be categorized as supervised or unsupervised. In supervised feature selection, such as filtering feature selection, features are selected based on their discriminative value of outcomes. These types of feature selection methods, however, are prone to over-fitting^15^ and ignore the effects of interaction of features among themselves which may benefit the predictive model. Furthermore, an individual feature alone may not be able to distinguish clinical outcome, but it may offer valuable information when combined with other features. Thus, in the filtering method, when selecting features, those valuable information is lost. In contrast, unsupervised feature reduction is based on dimensionally reduction algorithms, which maintains more information in the dataset, and hence, is robust to over-fitting^16^. As a result. unsupervised feature selection is not based on clinical outcomes (i.e., endpoints), and thus, the effect of interaction among features is maintained^1^. In this study, 5 unsupervised feature reduction methods were investigated including Principle Component Analysis (PCA), Independent Component Analysis (ICA), Zero Variance (ZV), Near Zero Variance (NZV), and Consensus Clustering (CC) combined with PCA^17^,^18^.

Among non-filtering feature selection methods, PCA is the most well-known approach that selects a small number of uncorrelated variables, called “principal components”, which could explain most of the variation in the data. ICA not only removes correlations among the variables, it also removes higher order dependence and thus, further reduces the number of features^19^. ZV and NZV methods remove the features with zero or near zero variance, respectively. CC is a consensus clustering method which clusters the features into clusters that have maximum intra-class redundancy and minimum inter-class correlation, and then, it picks a representative feature for each cluster. CC + PCA is the combination of consensus clustering and PCA within those clusters which has more than 2 features^17^. The performance of these feature reduction techniques along with no reduction and a filtered feature selection method (Wilcoxon test) was evaluated using 8 common machine learning classifiers as listed in Table 2. The predictive performance of non-filter reduction techniques was also compared with the Wilcoxon method.

Radiomics-based prognosis models utilize the quantitative imaging features to generate predictions, or in many cases, classifications for endpoint events such as “Recurrence” or “No Recurrence”. In machine learning, classification is considered as a supervised learning task of inferring a function from labeled training data^20^. The classification algorithm analyzes the training data and outcomes (labels), based on which it builds an optimized predictive model^21^. In our study, 8 classification algorithms were used which include Random Forest (RF), Generalized linear model (GLM), Support Vector Machine (SVM), Naïve Bayes (NB), Neural network (NNET), K-nearest Neighbor (KNN), Mixture Discriminant Analysis (MDA), and Partial Least Squares Generalized Linear Models (PLS), and their predictive performance was measured by Area Under Receiver Operating Characteristic ROC curve (AUC) via cross-validation^22^,^23^,^24^,^25^,^26^.

Table 2 summarizes the feature selection and classification methods used in this study.

### Subsampling

Many clinical outcomes have unbalanced ratio, which do not meet the assumptions of balanced endpoints for most machine learning-based predicative models^27^. To tackle this problem, 4 subsampling methods were investigated including down sampling, up sampling, Random Over Sampling Examples (ROSE), and Synthetic Minority Over-sampling Technique (SMOTE)^28^,^29^. Down sampling method down-samples the majority cases during model training while up sampling method up-samples the minority cases. These two methods are rather simple but either lose information or create a “non-universal decision region” since the data points that are created are in fact duplicates and are not able to help the prognosis model obtain more information. Synthetic Minority over Sampling Technique (SMOTE) is an enhanced sampling method in which, the computation for new synthetic sampling is based on Euclidian distance for variables. As a result, the synthetic cases will have attributes with values similar to the existing cases and not merely replications as oversampling does, thus, increasing the representation of the minority class in the resulting dataset while reflecting the structure of the original cases^28^,^30^. It has been shown that SMOTE is robust to the variation of unbalanced ratio with a variety of classifiers^28^. In this study, SMOTE was shown to outperform the other subsampling methods and thus, it was used to generate new synthetic cases and the AUCs of SMOTE boosted dataset were compared with those of the original dataset.

### Software Packages

Feature extract was done by ProCanVAS^12^ and Matlab 2015a. Statistical Analysis, model training, and validation was done in R 3.2.5 and caret package^18^.

## Results

### Prediction performance

**Figure 2:**
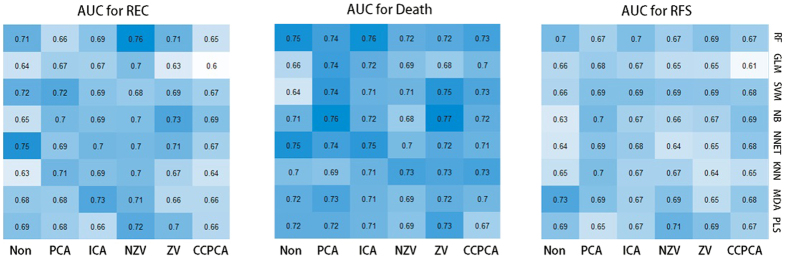
Individual results for 3 outcomes (recurrence (REC), Death, and recurrence free survival (RFS)).

Figure 2 shows the predictive performance of different combinations of classifiers and feature reduction methods for three clinical outcomes.

As it can be seen for recurrence (REC) in Fig. 2, the best result is achieved by RF classifier and NZV feature selection (AUC = 0.76). For death, NB classifier and ZV feature selection yield the highest AUC of 0.77. Finally, the best AUC for recurrence free survival (RFS) is achieved by MDA classifier with no need to use a feature section method (AUC = 0.73).

Averaging across different classifiers, among all feature selection methods, PCA method yielded the highest average AUC at 0.70 for all three outcomes. Although CC + PCA has shown its potential in feature selection, its prediction performance is not as high as other feature reduction techniques (AUC = 0.68). Considering its high computational cost and relatively low AUC, CC would not be the first choice as a feature selection techniques. While averaging all feature selection methods across all three outcomes, RF showed the highest mean AUC (0.71), which is consistent with previous results^4^. Many radiomics studies have used Support Vector Machine (SVM), which also shows relatively high prediction accuracy as well. Considering the general “Large P, small N” dataset where SVM works better than RF, SVM may also be taken into consideration when selecting proper classifiers for rather small datasets.

In this study, we used unsupervised feature selection methods to maintain more information in the dataset and achieve a competitive prediction accuracy. Previous research has shown that the WLCX feature selection method has high performance among supervised feature selection techniques^4^. To compare the performance of supervised and unsupervised feature selection methods, we compared the prediction performance of PCA and WLCX. The result showed that PCA (mean AUC = 0.70) has significantly higher AUC than that of WLCX which equals to 0.67 (P value = 0.0004643) indicating the potential in achieving higher prediction accuracy using unsupervised feature selection methods.

### Analysis of Variance

To investigate which factor significantly impacts the prediction performance measured by AUC, an analysis of variance was performed. In variance analysis, assumptions of ANOVA (i.e., normal distribution of residuals and equal variance in each group) were met. There were 48 cases for each feature selection method, 36 cases for each classification algorithm, and 96 cases for each endpoint.

**Figure 3:**
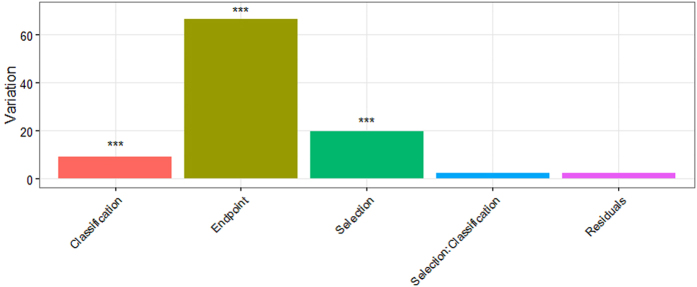
AUC Variance Analysis.

Through the analysis of variance, it is clear that different endpoints explained 65% of the variation in AUC. Feature selection techniques explained 20% and classification methods contributed another 10%. In ANOVA testing, these three factors have significant influence on AUC. However, the interaction of feature selection and classifiers is not significant and only explained less than 5% of the total variance, indicating that there is only minimal interaction effect between feature selection and classification methods. In other words, when choosing unsupervised feature selection methods and classifiers, there is no need to look at specific combination of selection method and classifier since the interaction is minimal (Fig. 3).

### Subsampling

The most unbalanced outcome data in our study was death with the ratio of 0.23. To investigate whether data balancing methods improve the results, we applied 4 subsampling methods to our death dataset namely down-sampling, up-sampling, ROSE, and SMPTE along with the original result with no subsampling using RF classifier. Although they improved AUC comparably, SMOTE was able to enhance AUC in a balanced way such that while maintaining high specificity, it was also able to increase sensitivity significantly, since the algorithm keeps more information without deleting or adding duplicate data entries.

**Figure 4:**
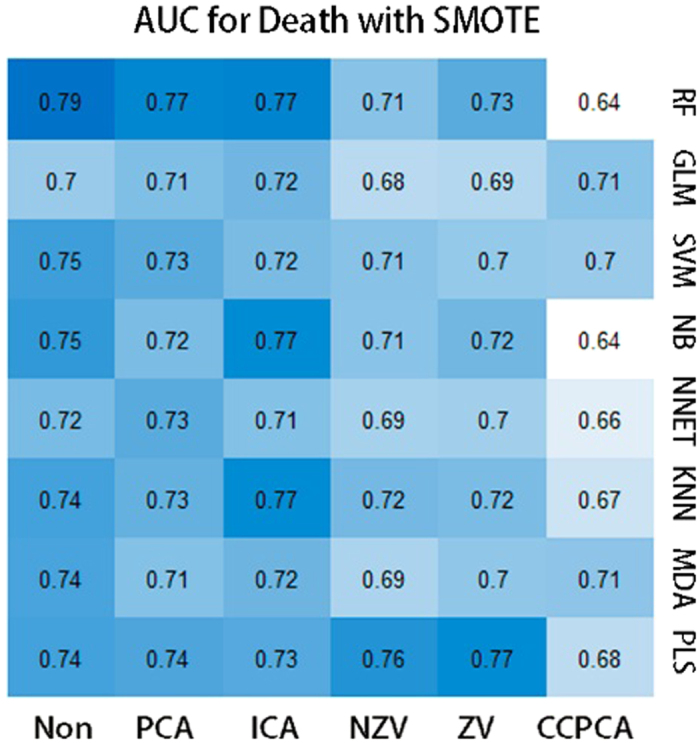
AUCs for Death data using SMOTE subsampling method.

Comparing the AUC for SMOTE and the original data for death outcome, applying SMOTE significantly improves AUC (P value = 0.03744), indicating the significant positive impact of SMOTE method on the prognosis performance. For best overall combination of classifier and feature selection (RF classifier and PCA feature selection), SMOTE increased AUC from 0.74 to 0.77 (Fig. 4).

### Sample Size

**Figure 5:**
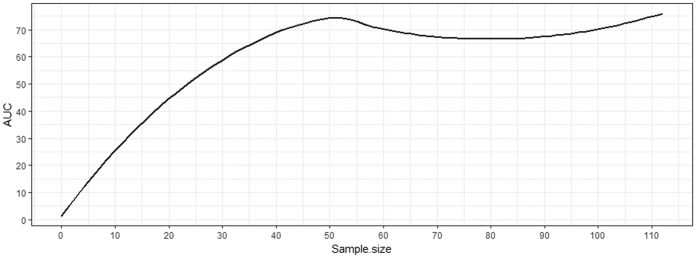
AUC for Death data for different sample sizes.

Sample size is an important factor in the predictive performance of radiomics-based prognosis. This is especially true for clinical studies where the sample size is often limited due to high cost of patient recruitment and collection of clinical outcome data. Furthermore, clinical outcomes often have unbalanced ratios, meaning that the observations for the minority class (e.g., death) are less frequent. This all leads to small sample sizes. On the other hand, it has been shown that machine learning algorithms generally need 80 to 560 observations to achieve a root-mean-square-error below 0.01^31^. To test the robustness of our radiomics-based prognosis analysis pipeline, an experiment was performed using the optimal pipeline (PCA + RF + SMOTE) for sample sizes ranging from 30 to 112 patients for the dataset used in this paper with death as outcome (Fig. 5).

It is interesting to observe that a threshold for the data size can be set (e.g., 50) below which, the predictive performance of radiomics-based prognosis may not be adequate. It is also worth noting that, as discussed in the previous section, subsampling methods such as SMOTE increase sample size by adding to the minority cases and thus, balancing the data. Therefore, with an outcome ratio of k (i.e., k = (# of majority cases)/(# of minority cases)), it can be shown that a subsampling method can increase the data size by a factor of 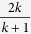. Thus, for our death dataset where the outcome ratio is 23% (i.e., k = 4.35), SMOTE increased the data size by ~63%. Hence, the lower-bound of sample size for our dataset (e.g., 50) is in fact increased to ~81, which is consistent with the one reported in the literature as the minimum number of cases needed for a robust predictive model^31^. It is seen that by increasing the sample size over 50, the AUC first drops slightly and then increases by adding more cases. The change in AUC is within the range (~3%) that could be observed when different cases enter the experiment randomly thus slightly affecting the performance negatively. As the number of cases increases, the AUC follows the overall trend of the plot; the more cases are added, a higher AUC is achieved.

The AUCs in Fig. 5 converge to ~0.70 when full sample set is used, which is lower than that reported in Fig. 4 (0.77 with PCA + RF + SMOTE for death). The reason for this apparent discrepancy is that for smaller data sizes (e.g., 30), some radiomic features had to be excluded since the classifier was unable to perform due to small sample size and high number of features. Thus, this experiment was performed with a subset of features leading to lower AUCs compared to the experiments with full dataset as reported in the Results section.

## Discussion

Radiomics has recently shown potential in achieving personalized medicine for different disease such as cancer. However, the large number of radiomic features, small number of observations, and unbalanced datasets are major challenges when building radiomics-based prognosis models. In this study, different unsupervised feature selection methods, classification techniques, and subsampling methods were investigated for different outcomes to study the optimal configuration and workflow for radiomic-based prognosis analytics.

Generally, a radiomics dataset contains a large number of features with a significant amount of noise and a highly correlated feature structure. These factors together are detrimental to the predictive model building process and negatively affect the prediction performance of the models. With similar data structure, genomics studies have overcome the feature redundancy problem through different feature selection approaches^32^,^33^. In radiomics domain, it is necessary to apply feature reduction when building robust prognosis models in order to achieve higher accuracy, better data visualizations and understanding, reducing measurement and storage requirements, and minimizing the training and inference time.

Among feature selection methods, the unsupervised feature selection, which is based on dimensionality reduction algorithms transforming the high-dimensional feature space into a meaningful representation^34^ was selected for our study. Compared to filtered (supervised) feature selection methods, the dimensionality reduction algorithms are less prone to over-fitting^16^. In our study, PCA showed great value in reducing the number of features, and yielded the highest overall (average) predictive performance. It is important to realize that some radiomic features may not have high predictive value individually but become important when interaction effects among the features are taken into consideration. Compared to filtered feature reduction techniques which may eliminate important features, unsupervised feature reduction maintains the interaction among features, benefiting the predictive model training process.

Consistent with previous results, RF has the highest predictive value among different classifiers. Using RF to build a predictive model with high specificity and sensitivity, it is also important to tackle the unbalanced data problem. Down-sampling, up-sampling, SMOTE and ROSE sub-sampling techniques were applied and showed improvement in terms of AUC, and especially sensitivity. For unbalanced dataset, machine learning algorithms tend to sacrifice the minority group to achieve a higher accuracy. Thus, in clinical radiomics studies, the subsampling methods can significantly improve the sensitivity, leading to better predictive performance. Although all these subsampling methods improved AUC comparably, SMOTE was able to enhance AUC in a balanced way; while maintaining high specificity, it was also able to increase sensitivity significantly. In addition, SMOTE has been shown to be robust to the variation of unbalanced ratio with a variety of classifiers^28^. Thus the combination of PCA feature selection, RF classifier, and SMOTE subsampling (PCA + RF + SMOTE) constitutes an optimal radiomics pipeline for prognosis of clinical outcomes. The analysis of variance also showed that while individual classifiers and unsupervised feature selection methods have significant impact on the prediction performance, the interaction effect of these two on the results is not significant. In other words, when choosing non-filtering feature selection methods and classifiers, there is no need to look at specific combination of selection methods and classifiers for high accuracy prediction model.

Although it has been previously reported in the literature that radiomics-based signatures may have a complimentary role in predicting survival and other clinical outcomes in early or advanced stage lung cancer patients^6^,^35^, none of these studies has specifically compared different data reduction methods and classification techniques, as done in this work, or addressed how the radiomics-based prognostic model can be optimized. Moreover, these studies focus on survival analysis using univariate or multivariate analysis where one or a few (e.g., 3) imaging features are used. In contrast, in this study, we take the predictive modeling approach where a large radiomics feature set is analyzed for prognosis of the disease. Nevertheless, the most similar study to this work is by Ganeshan *et al*.^6^ where the prognostic value of CT imaging features were studied for lung cancer. While we analyzed 3 outcomes (recurrence, death, and recurrence-free survival), Ganeshan *et al*. only analyzed one outcome namely death. Moreover, we used 112 cases compared to 56 in Ganeshan’s study^6^.

There are other related studies on lung cancer, all of which have used PET images^35^,^36^,^37^. Apart from the fact that in our study, we used CT images, all these studies only looked at one or found significant results for one clinical outcome (e.g., distant metastasis, death or disease-free survival respectively) while our study included 3 different clinical outcomes. Therefore, while these studies found prognostic value for only one outcome, in this paper, we show the prognostic value of an optimal radiomics-based pipeline for all 3 clinical outcomes (i.e., recurrence, death, and recurrence-free survival).

Survival analysis of clinical outcomes tend to focus on individual risk factors which influence the outcome the most. This has also been widely investigated in radiomics analytics to find significant features which influence a given clinical outcome the most. However, those significant features can only explain a small amount of variation individually, limiting their applications in clinical practice. Furthermore, since the number of features is usually high, survival analysis also faces the multiple testing problems. Thus, without proper control, survival analyses are expected to give a high amount of false positive significant features reducing the reliability of analysis. Thus, a feature-selection based analytics may mitigate this obstacle and help develop a more robust prognosis framework.

It has been previously shown that for NSCLC patients, radiomics-based predictive models can significantly improve the prognosis performance of traditional methods, which usually have rather low accuracy^36^. On the contrary, a well-trained radiomics-based prognosis model could make the prediction more accurate and unbiased. As more the potential of radiomics is realized, the number of radiomics datasets will increase significantly. How to fully take advantage of such data is a challenge in this field. Through this study, we aimed to determine the optimal configuration of radiomics-based predictive modeling analytics in terms of feature selection, subsampling, and classifiers to improve the prediction performance for further clinical applications.

## Conclusion

In this study, an optimal radiomics-based prognosis model for NSCLC patients for different outcomes were investigated. It was found that in order to achieve the optimum performance for predictive model, unfiltered feature selection methods such as PCA must be used in conjunction with sub-sampling techniques such as SMOTE and proper classification models such as Random Forest technique.

### Ethics Approval

The institutional research ethics board approved this retrospective single institution study.

## Additional Information

**How to cite this article**: Zhang, Y. *et al*. Radiomics-based Prognosis Analysis for Non-Small Cell Lung Cancer. *Sci. Rep.* **7**, 46349; doi: 10.1038/srep46349 (2017).

**Publisher's note:** Springer Nature remains neutral with regard to jurisdictional claims in published maps and institutional affiliations.

## Figures and Tables

**Figure 1:**
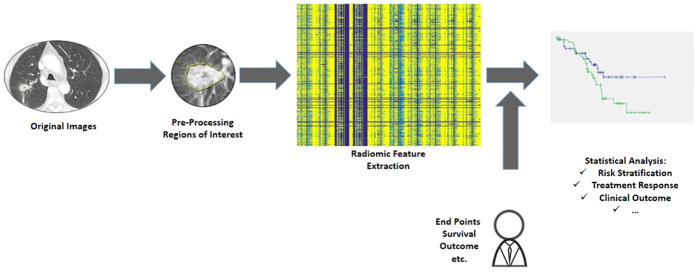
Radiomics Analytics Pipeline.

**Table 1:** Summary of radiomics features.

Feature group	Number of features	Description
Statistical - First order	11	ROI Size (# of pixels), ROI size (mm^2^), Mean gray level, Standard Deviation, Median gray level, Min ROI, Max ROI, Mean Positive Values, Uniformity, Kurtosis, Skewness
Textural - Second order	19	Contrast, Energy, Correlation, Homogeneity, Entropy, Normalized Entropy, Variance, Inverse Difference Moment, Sum of Average, Sum of Variance, Sum of Entropy, Difference of Variance, Difference of Entropy, Information Measure of Correlation, Autocorrelation, Dissimilarity, Cluster Shade, Cluster Prominence, Maximum Probability

**Table 2:** Summary of feature selection and classification methods.

Feature Reduction methods	Abbreviation	Classifiers	Abbreviation
No selection	NON	Random Forest	RF
Principal component analysis	PCA	Generalized linear model	GLM
Independent component analysis	ICA	Support Vector Machine	SVM
Near zero variance	NZV	Naïve Bayes	NB
Zero Variance	ZV	Neural network	NNET
Consensus Clustering + PCA	CC + PCA	k-nearest neighbor	KNN
Wilcoxon	WLCX	Mixture Discriminant Analysis	MDA
		Partial Least Squares GLM	PLS
